# Oral Candidal Load and Oral Health Status in Chronic Obstructive Pulmonary Disease (COPD) Patients: A Case-Cohort Study

**DOI:** 10.1155/2021/5548746

**Published:** 2021-09-11

**Authors:** Shahnawaz Khijmatgar, Giridhar Belur, Rajesh Venkataram, Mohmed Isaqali Karobari, Anand Marya, Veena Shetty, Avidyuti Chowdhury, Martin Gootveld, Edward Lynch, Sunena Shetty, Shilpa Shenoy, K Chaithra, Chitta Ranjan Chowdhury

**Affiliations:** ^1^NITTE (Deemed to be University), AB Shetty Memorial Institute of Dental Sciences, Department of Oral Biology and Genomic Studies, Mangalore, India; ^2^NITTE (Deemed to be university), KS Hegde Medical College and Hospital, Department of Pulmonary Medicine, Mangalore, India; ^3^Conservative Dentistry Unit, School of Dental Sciences, Universiti Sains Malaysia, Health Campus, 16150 Kubang Kerian, Kota Bharu, Kelantan, Malaysia; ^4^Department of Conservative Dentistry & Endodontics, Saveetha Dental College & Hospitals, Saveetha Institute of Medical and Technical Sciences University, Chennai, 600077 Tamil Nadu, India; ^5^Department of Orthodontics, Faculty of Dentistry, University of Puthisastra, Phnom Penh, Cambodia; ^6^NITTE (Deemed to be University), KS Hegde Medical College and Hospital, Department of Microbiology, Mangalore, India; ^7^Global Child Health, King's College London, UK; ^8^Health and Life Science, De Montfort University, The Gateway, Leicester LE1 9BH, UK; ^9^School of Dental Medicine, University of Nevada, Las Vegas, USA; ^10^The University of Bolton, City of London Dental School, Southgate Dental Science Campus, London, UK

## Abstract

**Objective:**

The objective of this study was to determine the candidal load of the patients with Chronic Obstructive Pulmonary Disease (COPD) and evaluate the oral health status of subjects with COPD. *Material and Methods*. *N* = 112 COPD subjects and *N* = 100 control subjects were included in the study. The selection of COPD cases was confirmed based on the set criteria from the American College of Physicians. The oral health status was assessed as per WHO criteria to determine the score of decayed, missing, and filled teeth (DMFT), significant caries index (SiC), community periodontal index and treatment needs (CPITN), and oral hygiene index-simplified (OHI-S). Gram staining was performed to identify Candida using the whole saliva. Quantitative evaluation of the candidal load was carried out using Sabouraud Dextrose Agar (SDA). Chrome agar was used to differentiate between the commensal carriages. A statistical analysis paired *t*-test and 95% confidence interval (CI) for proportions was carried out using STATA software.

**Results:**

Candidal growth was found in 21.42% (*n* = 24) of COPD cases and 1.1% (*n* = 11) of control cases (*p* < 0.05) (95% CI 0.45, 0.59). The DMFT score was 8.26 in COPD subjects and 4.6 in controls, the SiC score was 16.42 in COPD subjects and 10.25 in controls, and the CPITN score for both COPD and control cases was score 2.

**Conclusion:**

In conclusion, there was a higher candidal load among subjects suffering from COPD. Theophylline medication can be a risk factor for increased candidal load in COPD patients.

## 1. Introduction

Chronic Obstructive Pulmonary Disease (COPD) is a lung disease characterized by airway obstruction resulting in difficulty in breathing [1]. COPD is progressive, with symptoms ranging from severe cough, sometimes with the production of sputum; shortness of breath; wheezing; to tightness of the chest [2]. COPD is caused by long-term exposure to fume/smoke from the environment and impaired function of the lungs and the airways. The global prevalence of physiologically defined COPD in adults aged >40 years is approximately 9-10 percent [2]. In 2012, an Indian study on the epidemiology of asthma, respiratory symptoms, and chronic bronchitis in adults showed that the overall prevalence of chronic bronchitis in adults > 35 years was 3.49 percent [3, 4]. The most common cause was tobacco smoking (including second-hand or passive smoking) [1]. Other contributing factors included air pollution, primarily associated with poor ventilation whilst cooking with wood fires, including those who cook by using coal and biomass. Occupational exposures are believed to be the cause of 10-20% of COPD cases [1, 2, 5].

Inhalational therapy is the most employed method to treat COPD cases. However, studies revealed that the inhaled drugs used in the treatment of COPD have an adverse effect on oral health, and again, that is based on the dosage, frequency of exposure, and duration of use. Several oral conditions such as xerostomia, dental caries, candidiasis, mucosal ulceration, gingivitis, periodontitis, dry mouth, and taste changes have been associated with inhalational therapy [6]. For example, it has been established that prolonged use of *β*2-mimetic agents is associated with an increased rate of dental caries [7].

The impact of this study would help to learn more about the association of COPD cases and oral conditions like dental caries; periodontal diseases; gastrooesophageal reflux, including the high load of Candida; and increased risk of candidiasis among COPD cases. A study by Kowalski et al. [8] assessed that dry oral mucosa and dental caries are typical among COPD cases. The study had equal numbers of subjects (*n* = 100) in each group, i.e., for COPD and control. The rate of smoking was similar in both groups. The authors concluded that thrush appeared to be the most frequent oral ailment among COPD cases. Tooth loss and reduced teeth numbers were observed significantly more often in COPD patients than in the control group (*p* < 0.05). However, the frequency and type of caries did not differ significantly between the two groups (*p* > 0.05) [8]. The findings create evidence that the general dentists and other healthcare professionals are opted to manage the COPD cases, and they need to be aware of the risk development in terms of candidal infection in COPD cases [7, 8]. The rationale of this study is to explore any association between dental caries, periodontal diseases, dry mouth, and gastrooesophageal reflux with Candida among COPD patients of India, which has not been investigated combining oral health status and candidal load level. Hence, there is a need to carry out the study to explore the profile oral health status among Indian COPD cases. This study can develop recommendations and provide evidence-based information to the general dental practitioners for appropriate dental health care of the COPD cases visiting their dental practice.

The objective of this study was to determine the oral health status and candidal load in COPD compared to the control subjects.

## 2. Materials and Methods

### 2.1. Sampling

A sample size of control (*N* = 100) and COPD (*N* = 100) patients was needed to identify the candidal load among COPD patients and oral health with 80% power, using a two-sample *t*-test and assuming a (two-sided) *α* of 0.05 and *β* = 0.20.

### 2.2. Patients

Subjects of at least 18 years of age with American Thoracic Society (ATS)/European Respiratory Society- (ERS-) defined COPD for at least six months and who were current or former smokers (>10 pack-years) were selected. Patients with a current diagnosis of asthma or poorly controlled COPD were excluded. Current COPD therapy is maintenance therapy with ≥1 long-acting bronchodilator (LAMA, LABA, or inhaled corticosteroid (ICS)/LABA combination) for ≥4 weeks before screening [9]. The inclusion criteria, for control patients, was as follows: the patients selected from the dental hospital setting irrespective of being nonsmokers, ≥18years of age, and not suffering from any lung disease for the last 6 months. In addition, the patients with low mental capacity, pregnant patients, noncooperative patients, and immunocompromised patients were excluded from the study. All patients were provided with a written informed consent format.

### 2.3. Questionnaire

The pretested questionnaire was designed based on validated items. In addition, the items were included based on qualitative research [9–18]. The data collection was performed to record the medications, such as theophylline, salbutamol, and other relevant drugs to treat COPD cases. The data was recorded by maintaining the confidential issue and obtaining consent from the patients' cohorts and the normal subjects as the control group.

### 2.4. Endpoints and Assessments

The primary objective of this study was to assess the subjects' oral health status. Therefore, the primary endpoint was to record decayed, missing, and filled teeth (DMFT), significant caries index (SiC), community periodontal index treatment needs (CPITN) scores, and oral hygiene-simplified (OHI-S) index. The secondary objective was to quantify the candidal load among COPD subjects and identify the species. Therefore, the secondary endpoint was to quantify the Candida using Sabouraud Dextrose Agar and identify the type of Candida using chrome agar [13].

### 2.5. Data Collection

The COPD subjects (group 1) who visited the Department of Pulmonary Medicine at the K.S. Hegde Medical Academy Hospital were referred to the Department of Oral Biology and Genomic Studies to assess their oral health status and collection of whole saliva samples for Candida. In addition, demographic information of the subjects, including the history of present illness and personal habits (social history), was recorded. The same parameters were assessed among the control healthy matched subjects.

### 2.6. Method of Saliva Collection

The diagnosed cases of COPD were selected, and informed consent was sought as per the study protocol. They were adequately informed about the purpose of the study and examined and assessed on the same day. Patients were asked to provide the whole saliva spitting gently (without cough spattering) from the oral cavity for a minute, and the samples were collected in provided sterilized polyethylene containers. The containers were immediately transported to the Department of Microbiology of the K.S. Hegde Medical Academy to enumerate and assess Candida. The sample collection and transfer of the sample were within 30 mins of sample collection in the same campus of the hospital and the institute. Later, the saliva was stored in the incubator and transferred to the culture media.

### 2.7. Identification of Candida

Whole saliva was collected in the supplied specified container mentioned and in cases where the patient had difficulty spitting saliva. The materials used in the study include a Gram Stain Kit K001 by HiMedia for Candida detection by light microscopy. A smear on the glass slide was prepared and stained with the Gram stain for Candida detection. Quantitative estimation of the candidal load was carried out after using Sabouraud Dextrose Agar [13]. Chrome agar was used to differentiate between the commensal carriage and pathogenic coexistence of Candida in the oral cavity [13].

### 2.8. Statistical Analysis

Data was collected in an excel sheet, and statistical analysis was carried out using STATA (STATA-14, StataCorp LP). STATA was used to determine the descriptive statistics, risk ratio (RR), risk difference (attributable risk), odds ratio (OR), and 95% confidence intervals. It was planned that if the data was normally distributed, a parametric *t*-test would be used to establish significance, and if not, a normally distributed nonparametric test would be carried out.

### 2.9. Direct Microscopy

The morphological features of Candida species, including its shape, size, number of buds, attachment, thickness, and number of nuclei, were examined for identification. A smear was used to differentiate between yeast and hyphal forms and later confirmed by a culture method [13].

### 2.10. Laboratory Culture

*Swab collection*: the sampling approach involved gently rubbing a sterile cotton swab over the lesioned tissue and then subsequently inoculating the primary isolation medium, Sabouraud Dextrose Agar (SDA) [13].

Quantitative estimation of the fungal load was done by using chrome agar to differentiate between the commensal carriage and pathogenic existence of oral Candida, with higher loads considered likely in the latter. CHROMagar was used to simultaneously identify colonies of Candida albicans, C. krusei, and C. tropicalis [19].

## 3. Declaration of Helsinki and Ethical Approval

This study was carried out at the Department of Oral Biology and Genomic Studies, A.B. Shetty Memorial Institute of Dental Sciences, NITTE University, Mangalore, Karnataka, India, in collaboration with the Department of Pulmonary Medicine and Department of Microbiology of the K.S. Hegde Medical College and Hospital between 2016 and 2017. The study adhered to the guidelines of the declaration of Helsinki. Ethical approval was granted from the local ethical institutional committee of NITTE University before conducting the study (NU/CEC/sts-84/2015). The patients were informed about the study, and informed consent was taken.

## 4. Bias

Bias was minimized during all the study phases by standardized calibration of a laboratory method for Candida identification and enumeration. The dataset was collected and collated in an excel spreadsheet for analysis with standard deviation. The principal investigator, coinvestigator, and microbiologist were blinded during saliva collection, candidal load estimation, and the data analysis.

## 5. Results

**Figure 1:**
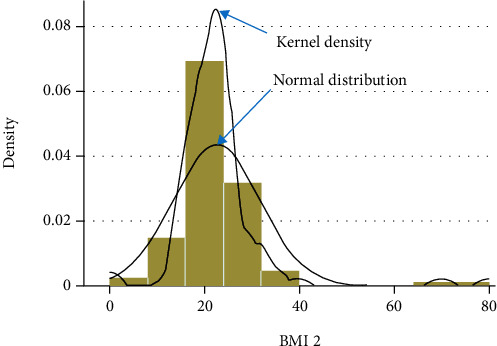
Normal distribution and kernel density.

**Figure 2:**
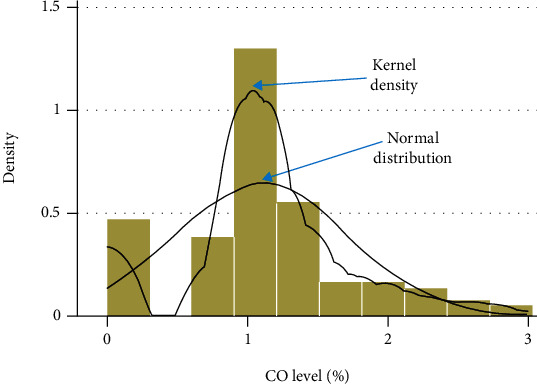
Normal distribution and kernel density.

The normality test distribution using kernel density is illustrated in Figures 1 and 2, and the data was not normally distributed. A sample size of group 1 is composed of subjects suffering from COPD (chronic bronchitis or emphysema) (*n* = 112), and for group 2, the control subjects' status (*n* = 100) was included in the study.

**Figure 3:**
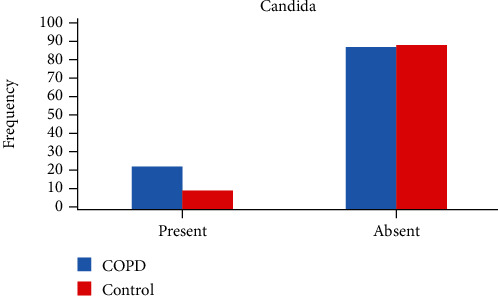
Candida growth detected in control and COPD subjects.

**Figure 4:**
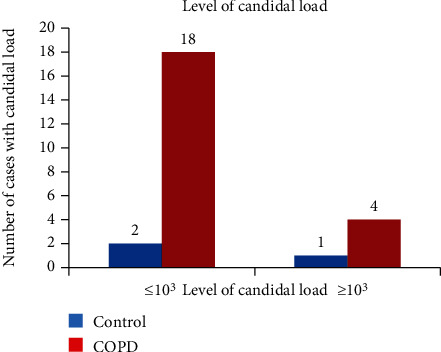
Level of candidal load in control and COPD subjects.

The demographics of COPD and control cases are demonstrated in Table 1. The average mean age of COPD subjects was 58.7 (26-80) years, and control was 41.66 (17-66) years. The individuals who had less education and worked as labourers and agriculturists were suffering more from COPD (Table 1). Candidal growth was found in 21.42% (*N* = 24) of COPD subjects and 1.1% (*N* = 11) of control cases, which was statistically significant (*p* < 0.01) (95% CI 0.45, 0.59) (Figures 1, 2, and 3). The level of candidal load among COPD cases was ≤10^3^ in *N* = 18 (Figure 4). The study results were similar to those found in previous studies [8]. The DMFT score, SiC, OHI-S score for COPD, and control cases are summarised in Table 2. The impact on oral health due to tooth loss is also established by a study done by Offenbacher et al. [12] and Kowalski et al. [8]. The caries prevalence did differ significantly between the COPD and control groups (Table 2) [8, 12].

The risk difference was 0.18 (0.018 and 0.35), risk ratio was 1.37 (1.05 and 1.80), and odds ratio was 2.20 (1.03 and 4.71) (*p* < 0.01). This is suggestive of the risk of increased candidal load among patients suffering from COPD. The CPITN scores for COPD and control cases were 2 in our study and the previous study [20].

## 6. Discussions

Generally, COPD predilection among males in India is high due to the consumption of smoking tobacco. Also, lower literacy rates and labour class individuals are most affected with COPD. This trend was seen in most noncommunicable diseases, and our results have found a similar trend. Socioeconomic status is the prime factor that leads to increased stress levels and leads to acquiring habits like smoking. In our COPD samples, the majority of the cases were heavy smokers (Table 1). WHO has laid directions on how to overcome socioeconomic status that would prevent unintended consequences.

The CPITN score 2 suggests bleeding on probing, pocket depth between 3.5 and 5.5 mm, overhanging restorations, and supra- and subgingival plaques. However, the score in both groups remains the same. The patients who were coming to the department of pulmonary medicine should have periodontal problems because they were on long-standing COPD and on medications that put them at risk of having periodontal disease (PD). The physicians have prevented this as they educate and counsel the COPD patients regarding the ill effects of the condition and the medication. The patients on inquiry replied that they brush twice daily, and after every use of an inhaler, they rinsed the mouth with water. Therefore, it is recommended for general dentists to educate and counsel COPD patients in their practices. This helps to bring down the periodontal problems among COPD patients.

However, studies like Scannapieco and Ho [21], Sharma and Shamsuddin [22], Hyman and Reid [23], Wang et al. [24], Liu et al. [25], Kucukcoskun et al. [26], and Ledic et al. [27] have found the difference in the occurrence of periodontal disease among COPD patients and control groups. These studies have established that COPD patients are more prone to periodontal disease than control groups. A systematic review by Scannapieco et al. [28] also found a strong correlation between PD and COPD cases. According to the meta-analysis by Zeng et al. [29], there was a publication bias among the studies included but a strong correlation between periodontal disease and COPD cases was found. Dental tissues are at an advantage because of their accessibility over internal organs. However, the diagnosis is mainly based on the clinical and radiographic examinations, making it more vulnerable to false assumptions and interpretation bias. Therefore, there is a need for a molecular method that can establish this relationship. A biomarker, i.e., a biological molecule used as an indicator for the substance or process of interest, can be used to establish this relationship. This helps in measuring the clinical parameters, behavior, or molecular/cellular endpoints. A study by Barros et al. [30] was aimed at analyzing the effect of periodontal disease, edentulism, and systemic biomarker inflammation in the occurrence of COPD. The levels of serum IL-6 in the highest two quartiles are at higher risk for COPD-related events. The molecular biomarkers serve as an alternative or adjunctive strategy for more accurate, sensitive, and specific diagnosis and prediction for prevention and outcome measurement.

A recent study in India by Raj et al. tried to determine the effect of 6 months of COPD medication on oral health parameters [31]. All but 1.2% of COPD and control cases did not use a toothbrush and toothpaste, and 55.5% of them brushed once a day. Ninety-seven percent of COPD cases were on inhalers, and the frequency of medication was twice a day among 88.4% of COPD patients [31]. The medication used was corticosteroids and long-acting beta-agonists (LABA) in 86.5% of cases. The DMFT, SiC, OHIS, gingival and periodontal problems, calculus, and plaque component of periodontal disease index (PDI) and colony-forming units (CFU) were significantly higher in COPD cases as compared to control cases [31]. Our study found that the DMFT and SiC were found to be higher in COPD cases, but the OHIS score for both groups was similar (Table 2). The possible explanation for the high DMFT rate and SiC score was that the patients underwent inhalational therapy and were on medications. This has led to the lower salivary flow rate and pH value along with the inhalation therapy [20, 32]. On the other hand, the OHIS score was fair because the doctors at the pulmonary medicine department were instructing patients to rinse the oral cavity after every use of the inhaler and maintain good oral hygiene. The subjects also confirmed the same when asked if they had rinsed the oral cavity after every inhaler use.

**Figure 5:**
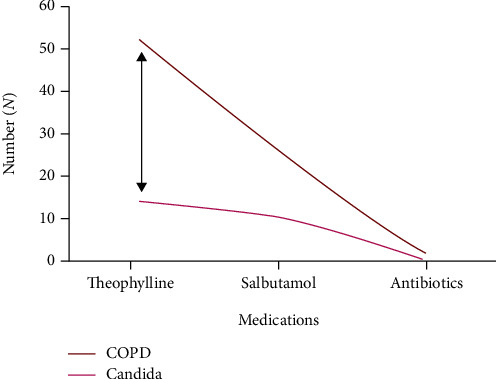
Schematic representation of the relationship between type of medication in COPD and its impact on the candidal load.

The most common approach used in treating COPD is by using bronchodilators. Bronchodilators are frequently used to treat COPD, and a recent study confirmed that their use has become excessive in treating COPD in general medical practice [33]. Also, most of the patients use inhalers without a spacer [34]. Similar findings were also found in our study (Table 2). Subjects who have been treated with corticosteroids and a combination of medicines suffer more from candidal infections (Figure 5) [10]. A study by Kowalski et al. [8] concluded that thrush appeared to be the most frequent oral cavity mucosa ailment in COPD patients. In our study, the patients were mostly on theophylline and salbutamol (Figure 5). More oral Candida was found among people who take theophylline in our study (Figure 5). According to the theophylline literature, the gender predisposition is for females, and more than 60-year-old subjects taking this drug for 1-6 months get more Candida [35]. There are no well-published previous reports on this novel finding. The most common acceptable biological mechanism is that theophylline has a side effect of causing gastritis, and later it leads to the development of gastroesophageal reflux. If the patients have a loose or open sphincter, the reflux leads to lower pH in the oral cavity. This, in return, puts the patients at risk for the increased candidal load. In our study, *N* = 42 patients of COPD were suffering from gastroesophageal reflux, gold standard II and III COPD cases. Out of *N* = 42, 10% had candidal load [36]. There was also dry mouth among *N* = 64 patients (Tables 1 and 3).

**Figure 6:**
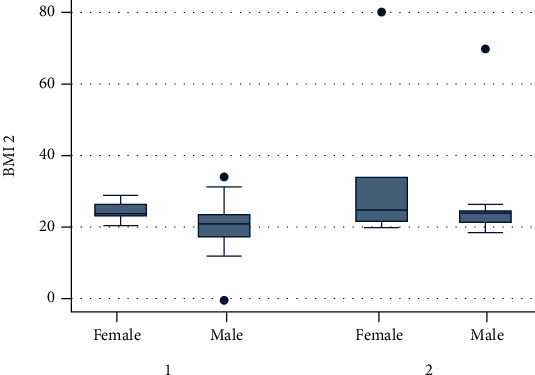
BMI: group 1 (COPD) and group 2 (control).

Przybyłowska et al. [10] aimed to assess the relationship between denture plaque biofilm and its impact on oral health in COPD patients. The results showed a greater frequency of prosthetic stomatitis complicated by mucosal infections in COPD patients than healthy patients (Figure 6) [10]. The reason for this may be that the COPD patients were on inhaled glucocorticoids and home oxygen therapy. As a result, there may be thinning of the mucosa and mouth dryness, creating an opportunity for colonization by fungal microorganisms. The presence of these fungal microorganisms can modulate the immune response of a host. The findings of Przybyłowska et al. [10] and other studies confirmed that the candidal prevalence among COPD patients and the control group amounted to 75.5% and 57%, respectively (10, 11). In our study, there were no denture wearers. Based on Przybyłowska et al. [10] and our findings, it can be concluded that COPD patients are at risk of fungal-induced oral disease and the risk increases when the patient is a denture wearer. Therefore, dentists should make an informed decision in COPD patients and approach them differently regarding treatment planning and education [10, 11]. Furthermore, it is more problematic for patients who are socially isolated or have poor mobility or dyspnoea to attend dental practice or hospitals [18]. Also, a study by Kucukcoskun et al. [26] has shown that an improvement in periodontal health can help prevent the repeated exacerbation of COPD because it has been established that there is an association between microorganisms involved in chronic periodontitis and repeated COPD exacerbations (Kucukcoskun, 2013) [26, 37–40].

In general, there are two interconnecting factors in our study, i.e., it is possible that the medications used could influence candida carriage and oral health. In addition, it is also possible that poor oral hygiene and oral health could influence COPD and Candida. The relationship and influence of these two factors on the candidal load among COPD cases and control in this study were well established in Figures 3, 4, and 5.

In our study, the samples were selected from the healthcare setting where the patients were coming for the consultation in the Pulmonary Medicine and Outpatient Department of the Dental Hospital. Therefore, the interpretation of the results should be made to keep selecting the sample factor and selecting samples in previous and future studies.

## 7. Conclusion

In conclusion, COPD patients had more caries and missing, decayed, and filled teeth and an increased candidal load compared to the control group. The oral hygiene status was fair in both groups. The CPITN scores were also similar for both the COPD and control patients. The candidal load among COPD patients can be lowered by advising patients to rinse the oral cavity after every bronchodilator use, especially theophylline. This study found that theophylline raises the candidal load compared to other drugs in patients suffering from COPD. Further, studies would help to make proper recommendations to the general practitioners and dentists.

## Figures and Tables

**Table 1:** Demographics of COPD and control subjects.

Variables		COPD (*N* = 112)	Control (*N* = 100)
Age (years)		58.7 (26-80)	41.66 (17-66)
Gender	Male	106	65
	Female	06	34
Education status	Illiterate	21	01
	Primary	53	07
	High school	30	32
	Preuniversity	03	04
	University	00	06
	Diploma	00	05
Occupation	Unemployed	08	12
	Labour	59	20
	Agriculture	23	01
	Service	07	40
	Business	08	10
Gastroesophageal reflux	Yes	42	03
	No	72	97
Dry mouth	Yes	64	07
	No	48	93

**Table 2:** Overview of the use of inhalers and its impact on candidal load.

Variables		COPD (*N* = 112)	Control (*N* = 100)
Use of inhaler	Yes	41	00
	No	57	99
Type of inhaler	Regular	38	—
	Spacer	03	—
Candidal load	Regular	12	—
	Spacer	1	—

**Table 3:** Oral health status of COPD and control cases.

Variables	COPD cases (*n* = 112)	Control cases (*n* = 100)	*p* < 0.05
Oral hygiene simplified index (OHIS)	1.82 (fair)	1.26 (fair)	NS
Decayed, missing, and filled teeth (DMFT)	8.26	4.6	<0.001
Significant caries index (SiC)	16.42	10.25	<0.001

NS: not significant.

## Data Availability

All associated data can be provided if needed.
